# The effectiveness of dentifrices without and with sodium lauryl sulfate on plaque, gingivitis and gingival abrasion—a randomized clinical trial

**DOI:** 10.1007/s00784-015-1535-z

**Published:** 2015-08-22

**Authors:** S. Sälzer, N.A.M. Rosema, E.C.J. Martin, D.E. Slot, C. J. Timmer, C. E. Dörfer, G.A. van der Weijden

**Affiliations:** Clinic for Conservative Dentistry and Periodontology; School for Dental Medicine, Christian-Albrechts-University Kiel, Kiel, Germany; Department of Periodontology, Academic Centre for Dentistry Amsterdam (ACTA), University of Amsterdam and VU University, Gustav Mahlerlaan 3004, 1081 LA Amsterdam, The Netherlands; Sara Lee Corporation, Amersfoort, Netherlands

**Keywords:** Gingivitis, Plaque, Gingival abrasion, Manual toothbrush, Dentifrice, Toothpaste, Sodium lauryl sulfate, SLS

## Abstract

**Objectives:**

The aim of this study was to compare the efficacy of a dentifrice without sodium lauryl sulfate (SLS) to a dentifrice with SLS in young adults aged 18–34 years on gingivitis.

**Material and methods:**

One hundred twenty participants (non-dental students) with a moderate gingival inflammation (bleeding on probing at 40–70 % of test sites) were included in this randomized controlled double blind clinical trial. According to randomization, participants had to brush their teeth either with dentifrice without SLS or with SLS for 8 weeks. The primary outcome was bleeding on marginal probing (BOMP). The secondary outcomes were plaque scores and gingival abrasion scores (GA) as well as a visual analogue scale (VAS) score at exit survey. Baseline and end differences were analysed by univariate analysis of covariance (ANCOVA) test, between group differences by independent *t* test and within groups by paired sample *t* test.

**Results:**

BOMP improved within groups from on average 0.80 at baseline to 0.60 in the group without SLS and to 0.56 in the group with SLS. No statistical difference for BOMP, plaque and gingival abrasion was found between both groups. VAS scores for taste, freshness and foaming effect were significantly in favour of the SLS-containing dentifrice.

**Conclusion:**

The test dentifrice without SLS was as effective as a regular SLS dentifrice on gingival bleeding scores and plaque scores. There was no significant difference in the incidence of gingival abrasion.

**Clinical relevance:**

In patients diagnosed with gingivitis, a dentifrice without SLS seems to be equally effective compared to a dentifrice with SLS and did not demonstrate any significant difference in gingival abrasion. In patient with recurrent aphthous ulcers, the absence of SLS may even be beneficial. However, participants indicate that they appreciate the foaming effect of a dentifrice with SLS more.

## Introduction

The surfactant (or detergent) is an agent which is added to a dentifrice in order to exert cleansing and antibacterial effects through a surface action, depending on hydrophilic and hydrophobic properties [1]. The most widely used surfactant in dentifrices is sodium lauryl sulfate (SLS) (C_12_H_25_NaO_4_S) which has been used for more than 50 years [2]. SLS is the sodium salt of lauryl alcohol (1-dodecanol). It is designated as sulfuric acid monododecyl ester sodium salt. The most common used concentrations vary from 0.5–2 % [2]. However, some manufacturers have moved away from SLS and introduced other, less irritant surfactants. Dentifrices such as Zendium® (Sara Lee, Amersfoort, The Netherlands) do not contain SLS but contain alternatively less irritant surfactants like non-ionic polyethylene glycol ethers of stearic acid (e.g. stearyl ethoxylate (30) EO). Toothpastes containing amine fluoride such as olaflur typically do not contain added surfactants as the amine cation functions as surfactant molecule.

Besides the enhancement of the foaming effect, surfactants are thought to reduce the surface tension which also creates the impression of cleanliness [3]. The surfactant also aids in the intra-oral dispersion of toothpaste and in the micellization of hydrophobic ingredients, such as flavour compounds and antiplaque/antigingivitis actives [4]. Furthermore, SLS inhibits the growth of a number of microorganisms. The antimicrobial action of SLS is related to its adsorption and penetration through the porous cell wall followed by interaction with components of the cell membrane, lipids and proteins. The penetration of SLS into the membrane causes an increase in cell permeability of the bacteria, which may result in leakage of intracellular components and cell lysis [5]. These ‘in vitro’ data are supported by clinical results from studies on 1–1.5 % SLS mouth rinse. These studies demonstrated plaque inhibition following twice daily usage [6–9]. According to a study by Landa et al. [10] SLS might penetrate deeply into biofilms.

Besides these beneficial effects, SLS might have a negative influence in patients with recurrent aphthous ulcers (RAU) [11, 12, 4]. The mechanism by which SLS induces oral mucosal desquamation is probably multi-factorial, due to the surface active nature of this molecule. An SLS-induced elimination of the protective mucin surface layers may reduce the resistance of the oral mucosa [13]. A relationship has been shown between increased oral desquamation and the use of different brands of commercially available dentifrices [14–16]. In the study by Shim et al. [4] the duration of ulcers and mean pain score were significantly decreased during the period using SLS-free dentifrice compared to two SLS-containing dentifrices (1.5 %). The leading toothpaste manufacturers still continue to utilize SLS because of its desired foaming ability, acceptable taste and low cost in relation to other surfactants. Only very few currently marketed toothpastes contain a surfactant other than SLS [17].

With an obvious discrepancy between the beneficial efficacy and potential side effects, the aim of this study was to assess the effect on gingivitis and plaque scores of a dentifrice without SLS compared to a standard dentifrice with SLS.

## Material and methods

### Ethical aspects

The study protocol was approved by the Medical Ethics Committee of the Academic Medical Centre in Amsterdam (MEC # 06/233). All voluntary participants were informed of the outline, purpose and duration of the study and signed an informed consent form before enrolment.

### Study population

**Fig. 1:**
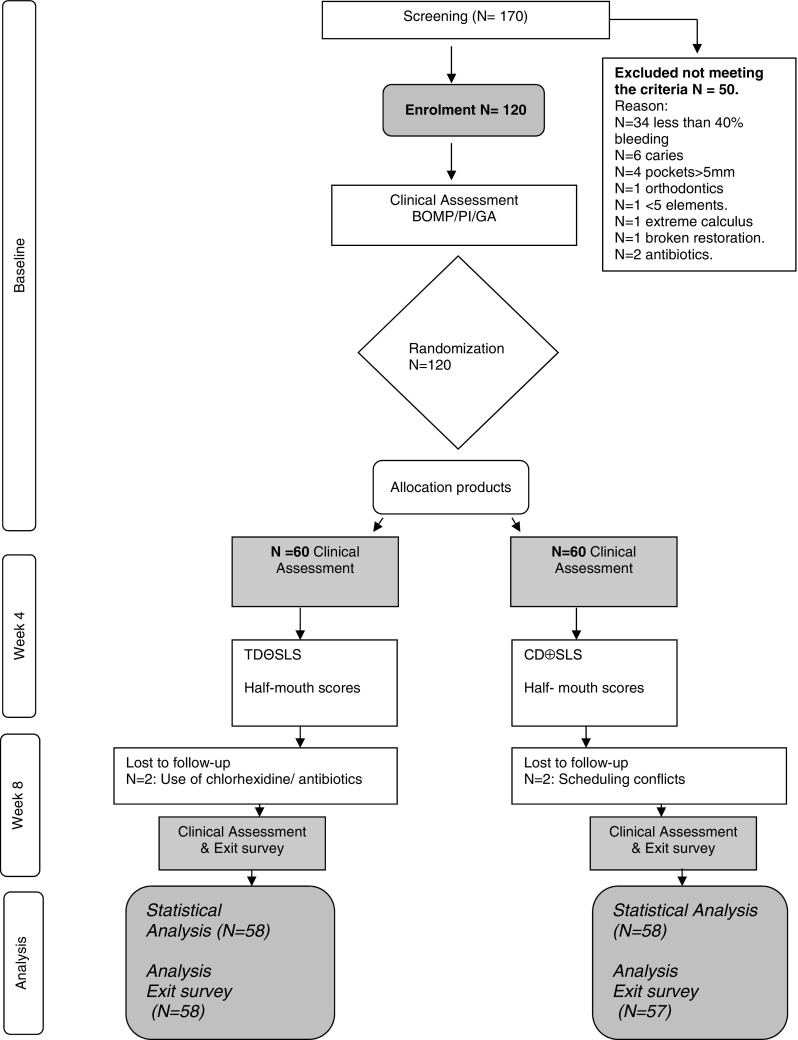
Flowchart depicting subject enrolment and measurements

The participants were non-dental students from University colleges in and around Amsterdam. They were recruited by email and flyer advertisement. One hundred and seventy adult participants in good general health were screened out of which 50 were rejected because they did not meet the inclusion criteria (see Fig. 1). Participants had to demonstrate at least five evaluable teeth in each quadrant and gingival bleeding of at least 40 %. A total of 120 participants were enrolled into this study. The sample size of 60 per group was calculated a priori in such a way that with an alpha of 0.05, a difference of 0.18 (between groups) of the bleeding index can be identified with 80 % power, based on a pooled standard deviation (SD) of 0.34 derived from a previous study [18].

### Study design

Eligible participants were randomly assigned to one of the two experimental groups of 60 participants each. Randomization was performed using generated random numbers (www.random.org). Allocation concealment was held by the dentifrice manufacturer. All products were packed in identical white tubes and were labelled by subject identification number. Participants received a manual multi-tufted soft toothbrush (filament 6–12 Nylon soft 6 mills, soft heart white outer setting, concave profile cutting centre lowest; Zendium Soft adult toothbrush). The participants received one of two commercially available dentifrices, the test dentifrice [TD ⊖ SLS] without SLS, the control with dentifrice [CD ⊕ SLS]. The TD ⊖ SLS dentifrice contained sodium fluoride 1100 ppm and the surfactant stearyl ethoxylate (30) EO (Zendium® classic, Sara Lee, Amersfoort, The Netherlands). The CD ⊕ SLS dentifrice contained fluoride sodium monofluorophosphate 1000 ppm/sodium fluoride 450 ppm and the surfactant sodium lauryl sulfate 1–5 % (Colgate® caries protection, Colgate-Palmolive Co., New York, NY, USA) [19]. The relative dentine abrasion (RDA) of approximately 60–70 was of similar range for both tested dentifrices [20].

All participants were provided with sufficient amount of their assigned products. They were asked to brush for the total duration of the study twice daily for 2 min, using only their provided toothbrush and assigned dentifrice. The use of any other dental products or interdental cleaning aids during the study was not allowed. Participants were instructed not to brush their teeth the evening before the clinical assessment to allow for scoring of overnight plaque accumulation.

### Parameters

This 8-week study was a double blind, randomized, controlled clinical trial with a 4-week intermediate assessment using a half mouth score design [21] randomized at baseline. Two contralateral quadrants were scored being the 1st and 3rd quadrant or the 2nd and 4th quadrant depending on randomization. The following indices were performed at baseline and at the 4 and 8 weeks evaluation. All measurements were performed by one blinded examiner under the same conditions (NAMR).

The level of gingival inflammation was the primary outcome of the study and was assessed according to the bleeding on marginal probing (BOMP) index as described by Van der Weijden et al. [22] and Lie et al. [23]. Bleeding was elicited with a WHO-approved ball-ended probe (Ash Probe EN15, DENTSPLY International, York, PA, USA). In brief, the gingival margin was probed at an angle of approximately 60° to the longitudinal axis of the tooth. The absence or presence of bleeding was scored within 30 s of probing on a scale of 0–2 (0 = non-bleeding, 1 = pinprick bleeding, 2 = excess bleeding) [22, 24].

Secondary outcomes were the plaque scores and gingival abrasion scores and the response to a questionnaire using visual analogue scales (VAS).

The plaque was measured according to the Modified Quigley and Hein index at six sites per tooth on a six-point scale (0–5) as described in detail by Paraskevas et al. [25].

Gingival abrasion sites were stained by applying Mira 2-Ton blue ® on the gingiva as proposed by Danser et al. [26]. The abrasions were stained blue and measured by using a PQ-William’s periodontal probe placed across the long axis of the lesions. Abrasions were scored as “small” if ≤2 mm, as “medium” if ≥3 but ≤5 mm and as “large” if >5 mm as proposed by [27]. Those lesions measuring between 2 and 3 mm were assigned a score of small or medium according to nearest mm mark on the probe.

Participants were asked to fill out an exit survey at the end of the trial period to assess their attitude to the assigned dentifrice and toothbrush. Visual analogue scales (VAS) [28] were used in the majority of questions to assess the participants’ opinions (see Table 1). Participants were requested to mark a point on a 10-cm-long uncalibrated line of which the two ends annotated with each of the extremes of each query, the left being the negative, and the right being the positive extreme.

**Table 1:** Subjects demographics and group assignment

Groups	Test dentifrice without SLS, [TD ⊖ SLS]	Control dentifrice with SLS, [CDS ⊕ SLS]
*N*	58	58
Female	50 (86 %)	41 (71 %)
Male	8 (14 %)	17 (29 %)
Mean age in years (standard deviation; range)	21.33 (SD 2.53; 18–29)	21.76 (SD 3.59; 18–34)
Brand	Zendium® classic	Colgate® caries protection
Contents	Sodium fluoride 1100 ppm Surfactant stearyl ethoxylate (30) EO RDA ± 60	Fluoride sodium monofluorophosphate 1000 ppm Sodium fluoride 450 ppm Surfactant sodium lauryl sulfate 1.5 % RDA ± 70

*RDA* relative dentin abrasion score

### Statistical analysis

Means and standard deviations for bleeding, plaque and gingival abrasion for all complete cases were calculated and analysed using SPSS 21 software [29]. Overall scores were compared with a univariate analysis of covariance (ANCOVA) test with baseline measurements as covariate and week 8 as dependent variable. An independent *t* test was used to test for statistically significant differences between groups at each time point. Differences within the groups were analysed using the paired sample *t* test, and confidence intervals were generated; visual analogue scale outcomes were analysed with an independent *t* test.

## Results

The 120 enrolled subjects were aged between 18 and 34 years (Table 1). During the study, there were four dropouts due to scheduling conflicts and medical problems unrelated to the study. In total, 116 participants completed the protocol. Eventually, 58 participants both in test and control group provided a full set of evaluable clinical data. The means and statistics were based on 116 participants that completed the protocol as shown in Fig. 1. Because one participant failed to fill out the exit survey, 115 participants completed the questionnaire.

The bleeding score did not differ significantly between groups at baseline, 4 weeks nor at 8 weeks (Table 2). The difference in mean bleeding scores within each group from baseline to 8 weeks shows a statistically significant reduction for both groups (*P* < 0.001).

**Table 2:** Mean (standard deviation) total bleeding scores [24] and total plaque scores [25]. Half mouth scores were performed as described by Bentley & Disney [21]. Overall statistics show no significant difference between both groups. Paired sample *t* test shows a statistical significant difference for bleeding on marginal probing within the groups

		Baseline	Week 4	Week 8	Diff (base‐8 weeks)	Statistics within groups 0–8 weeks^a^	ANCOVA^b^
Bleeding scores	TD ⊖ SLS (*N* = 58)	0.80 (0.19)	0.64 (0.18)	0.60 (0.23)	0.20 (0.22)	*P* < 0.001	*P* = 0.347
	CD ⊕ SLS (*N* = 58)	0.80 (0.19)	0.64 (0.19)	0.56 (0.24)	0.24 (0.23)	*P* < 0.001	
	*P* values analysis between groups^c^	*P* = 0.923	*P* = 0.959	*P* = 0.375	*P* = 0.403		
	95 % CI	−0.07; 0.07	−0.07; 0.07	−0.05; 0.12	−0.48; 0.12		
Plaque scores	TD ⊖ SLS (*N* = 58)	2.05 (0.47)	1.96 (0.38)	1.88 (0.36)	0.18 (0.36)	*P* < 0.001	*P* = 0.690
	CD ⊕ SLS (*N* = 58)	1.99 (0.46)	1.90 (0.41)	1.82 (0.42)	0.17 (0.31)	*P* < 0.001	
	*P* values analysis between groups^c^	*P* = 0.437	*P* = 0.414	*P* = 0.406	*P* = 0.912		
	95 % CI	−0.10; 0.24	−0.09; 0.21	−0.08; 0.20	−0.13; 0.12		

*95 % CI* 95 % confidence interval

^a^Paired Sample *t* test

^b^ANCOVA (baseline as covariate and week 8 as dependent variable)

^c^Independent *t* test

Correspondingly, no significant difference in plaque scores between TD ⊖ SLS group and CD ⊕ SLS were observed at any time point (Table 2). Within both of the groups, plaque scores reduced significantly during the study (*P* < 0.001).

The overall analysis on gingival abrasions showed no difference within the groups at any time point as shown in Table 3 There was no statistically significant difference between both groups for overall abrasions. Most (94 %) of the abrasions were small in size and therefore a sub-analysis was performed for abrasions sites ≤2 mm as shown in Table 3. Small abrasions between both groups showed no statistical significant difference at any time point. Regarding the taste, freshness and the foaming effect, a significant difference between both groups was observed (Table 4).

**Table 3:** Mean (standard deviation) total gingival abrasion sites: overall analysis including small (2 ≤ mm), medium (≥3 mm − ≤ 5 mm) and large (>5 mm) lesions; sub-analysis including small (≤2 mm) lesions. Half-mouth scores were performed as described by Bentley & Disney [21]. Overall statistics show no differences between both groups for each visit. Within groups difference (paired sample T tests) were statistical significant within groups for total abrasions and within neither group for small abrasions

		Baseline	Week 4	Week 8	Diff (base‐8 weeks)	Statistics within groups 0–8 weeks^a^	ANCOVA^b^
Gingival abrasions Overall	TD ⊖ SLS (*N* = 58)	4.72 (5.11)	5.36 (4.84)	5.39 (5.29)	−0.67 (5.67)	*P* = 0.370	*P* = 0.706
	CD ⊕ SLS (*N* = 58)	5.60 (5.32)	4.03 (4.17)	5.30 (4.09)	0.31 (6.32)	*P* = 0.710	
	*P* values analysis between groups^c^	*P* = 0.366	*P* = 0.116	*P* = 0.906	*P* = 0.380		
	95 % CI	−2.80; 1.04	−0.33; 2.99	−1.64; 1.84	−3.20; 1.23		
Gingival abrasions Small	TD ⊖ SLS (*N* = 58)	4.47 (4.86)	4.79 (4.43)	5.19 (5.13)	0.72 (5.43)	*P* = 0.315	*P* = 0.811
	CD ⊕ SLS (*N* = 58)	5.10 (4.92)	3.78 (3.77)	5.16 (3.93)	0.05 (5.97)	*P* = 0.948	
	*P* values analysis between groups^c^	*P* = 0.484	*P* = 0.186	*P* = 0.968	*P* = 0.527		
	95 % CI	−2.44; 1.16	−0.50; 2.53	−1.65; 1.72	−1.43; 2.77		

*95 % CI* 95 % confidence interval

^a^Paired sample *t* test

^b^ANCOVA

^c^Independent *t* test

**Table 4:** The outcomes presented in mean and (standard deviation) of the questionnaire were analysed with an independent *t* test to calculate the mean difference between groups, based on the VAS [28] with extremes to very unpleasant and very pleasant (from 0 to 10)

Question			With extremes		TD ⊖ SLS group *N* = 58	CD ⊕ SLS group *N* = 57	*P* value [95 % confidence interval of the difference between groups]
			From (0)	To (10)			
Taste perception		How was the taste of the dentifrice?	Very unpleasant	Very pleasant	5.40 (1.90)	6.87 (1.74)	*P* < 0.001 −2.15, −0.80
Freshness of the dentifrice	A	What is your opinion about the freshness of the dentifrice?	Stale	Fresh	4.64 (2.49)	6.29 (2.00)	*P* < 0.001^a^ −2.49; −0.82
	B	Did you find it….?	Not fresh at all	Too fresh	3.49 (1.71)	4.45 (1.37)	*P* = 0.001^a^ −1.53; −0.39
Duration of taste		How long did the taste remain?	Very short	Very long	3.64 (1.45)	3.90 (1.49)	*P* = 0.347^a^ −0.80; 0.28
Alteration of the taste sensation		How was your taste of food and drinks affected?	Negative change	Positive change	4.85 (1.11)	4.38 (1.59)	*P* = 0.066^a^ −0.03; 0.98
Foaming effect		What do you think about the foaming effect?	Too little foam	Too much foam	4.24 (1.62)	5.72 (2.04)	*P* < 0.001^a^ −2.16; −0.80

^a^Independent *t* test

## Discussion

The aim of this study was to assess the efficacy of a dentifrice without SLS in comparison to a dentifrice containing SLS as a surfactant. The alternative hypothesis that was used for this study assumed that there was a statistically significant difference between the efficacy of TD ⊖ SLS and CD ⊕ SLS. To the best of our knowledge, this is the first efficacy study that compared an SLS-free dentifrice containing stearyl ethoxylate (30) EO as a surfactant to an SLS-containing dentifrice. Neither the primary parameter of bleeding nor the secondary parameter of plaque showed statistically significant differences between the two groups of this study. Correspondingly, no significant difference in side effects as assessed by scoring the number of gingival abrasion sites was observed. However, the present study showed that the participants in the CD ⊕ SLS group who used the surfactant SLS where more satisfied about the taste, the freshness and the foaming effect than those participants in the TD ⊖ SLS group.

In clinical studies oral epithelial sloughing, ulcerations and inflammation caused by SLS have been observed [30, 31, 16]. The impairment of the barrier function of oral mucosa by denaturing the glycoproteins of the mucin layer through SLS dentifrice might lead to a higher vulnerability of the gingival and buccal mucosa to irritants such as exogenous antigens [3, 13]. The elimination of the mucin layer can be mediated by the calcium binding capacity of SLS molecules [3]. Mucin is the principal organic constituent of mucus, the visco-elastic material that covers all mucosal surfaces, and plays an essential role in the non-immune protection of the mucosal surfaces [32]. Surfactants might be responsible for a reduction in the level of keratinization of the human oral epithelium, probably due to rupture of the intercellular junction [33]. Widening of the stratum corneum because of separation and loss of surface epithelial layers by SLS has also been observed in an experimental model [2].

Thus, SLS is believed to increase the incidence of recurrent aphthous ulcers [2, 12, 13] by disintegration of the mucin layer, denaturation of proteins in the epithelial cells, solubilization of structural lipids of the cells and, finally, penetration of SLS into deeper layers of the mucosa where functions of the living tissue may be compromised [2]. The sensitivity to low concentrations of SLS was much higher for the oral mucosa than for the skin in animal models [34]. It is well known that SLS is an irritant to skin at high concentrations and that repeated application results in a dose-dependent contact dermatitis [35, 36]. According to the American College of Toxicology, in products intended for use on the skin and for prolonged contact, the concentration of SLS should not exceed 1.0 % [37]. However, in the case of the oral cavity, prolonged contact does not occur since the mucosal tissues are constantly being bathed by saliva. It has been shown that very little SLS is retained in the oral cavity. In the case of tooth brushing, approximately 9.6 mg SLS is retained after 20 min. The total area of the oral cavity is approximately 210 cm^2^; hence, it can be deduced that the maximum amount of SLS bound per cm^2^ would be 0.046 mg, a level at least 100-fold below that which is needed to cause irritation [38].

Although SLS is the most frequently used surfactant worldwide, there are alternatives that have less side effects such as stearyl ethoxylate or Alkyoamidobetaines [2]. As dentifrices with and without SLS clinically showed no difference in the present study with regard to gingivitis and plaque, an SLS-free dentifrice could be recommended to patients suffering from recurrent aphthous ulcers. Whether the frequency of recurrent aphthous ulcers is reduced when people start to brush with an SLS-free dentifrice remains still part of a discussion [11, 12, 4]. The reduction of aphthous ulcers might be dependent on the different brands of commercially available SLS-free dentifrices [14–16]. SLS-free dentifrice does seem to affect the ulcer healing process and reduces pain caused by the aphthous ulcers in daily lives of susceptible patients [4, 2].

Another side effect that has been reported is a reduced perception of taste after rinsing with a 1 % SLS solution, which can last for up to 4 h [7]. The turnover of taste cells alters and the SLS interferes with the inner mechanisms of the taste cells [39]. This has also been described as the ‘orange juice effect’. That is, when consuming a citrus juice drink just after toothbrushing with an SLS dentifrice, SLS causes an astringent and unpleasant taste sensation.

Local physical trauma may initiate ulcers in susceptible people [40, 41]. The present study therefore also investigated whether SLS influences the susceptibility to gingival abrasions following toothbrushing. No difference was found in the incidence between brushing without or with an SLS-containing dentifrice. These findings are in further support of the conclusion by Versteeg et al. [42] that dentifrice does not contribute to increased post-brushing gingival abrasion. In their study, they used a non-SLS dentifrice and found no difference in the incidence of gingival abrasion between bushing with or without a dentifrice.

However, in order to gain more representative data concerning the effectiveness of dentifrices without SLS, further long-term studies preferably of at least 6-months duration evaluating various dentifrices not containing SLS are needed.

## Conclusion

The test dentifrice without SLS was as effective as a regular SLS dentifrice on gingival health and plaque index scores. In addition to the expected foaming effect, an SLS-containing dentifrice was also significantly more appreciated toward taste perception, freshness and duration of taste.
